# Multimodality imaging: Bird’s eye view from The European Society of Cardiology Congress 2018 Munich, August 25–29, 2018

**DOI:** 10.1007/s12350-018-01517-7

**Published:** 2018-11-20

**Authors:** Victoria Delgado, Chiara Bucciarelli-Ducci, Oliver Gaemperli, Pal Maurovich-Horvat, Jeroen J. Bax

**Affiliations:** 1 Department of Cardiology, Heart Lung Centrum, Leiden University Medical Center, Albinusdreef 2, 2300 RC Leiden, The Netherlands; 2 Bristol Heart Institute, National Institute of Health Research (NIHR), Biomedical Research Centre, University Hospitals Bristol and University of Bristol, Bristol, UK; 3 HeartClinic Hirslanden, Zurich, Switzerland; 4 MTA-SE Cardiovascular Imaging Research Group, Heart and Vascular Center, Semmelweis University, Budapest, Hungary

**Keywords:** Echocardiography, computed tomography, nuclear imaging, cardiovascular magnetic resonance

## Abstract

At the European Society of Cardiology (ESC) congress of this year 2018, held in Munich from August 25th to 29th, 4594 abstracts were presented. Of those, 423 (10.8%) belonged to an imaging category. Experts in echocardiography (VD), cardiovascular magnetic resonance (CMR) (CBD), nuclear imaging (OG), and cardiac computed tomography (CT) (PMH) have selected the abstracts in their areas of expertise that were of most interest to them and are summarized in this bird’s eye view from this ESC meeting. These abstracts were integrated by one of the Editors of the Journal (JB).

## Echocardiography

**Figure 1:**
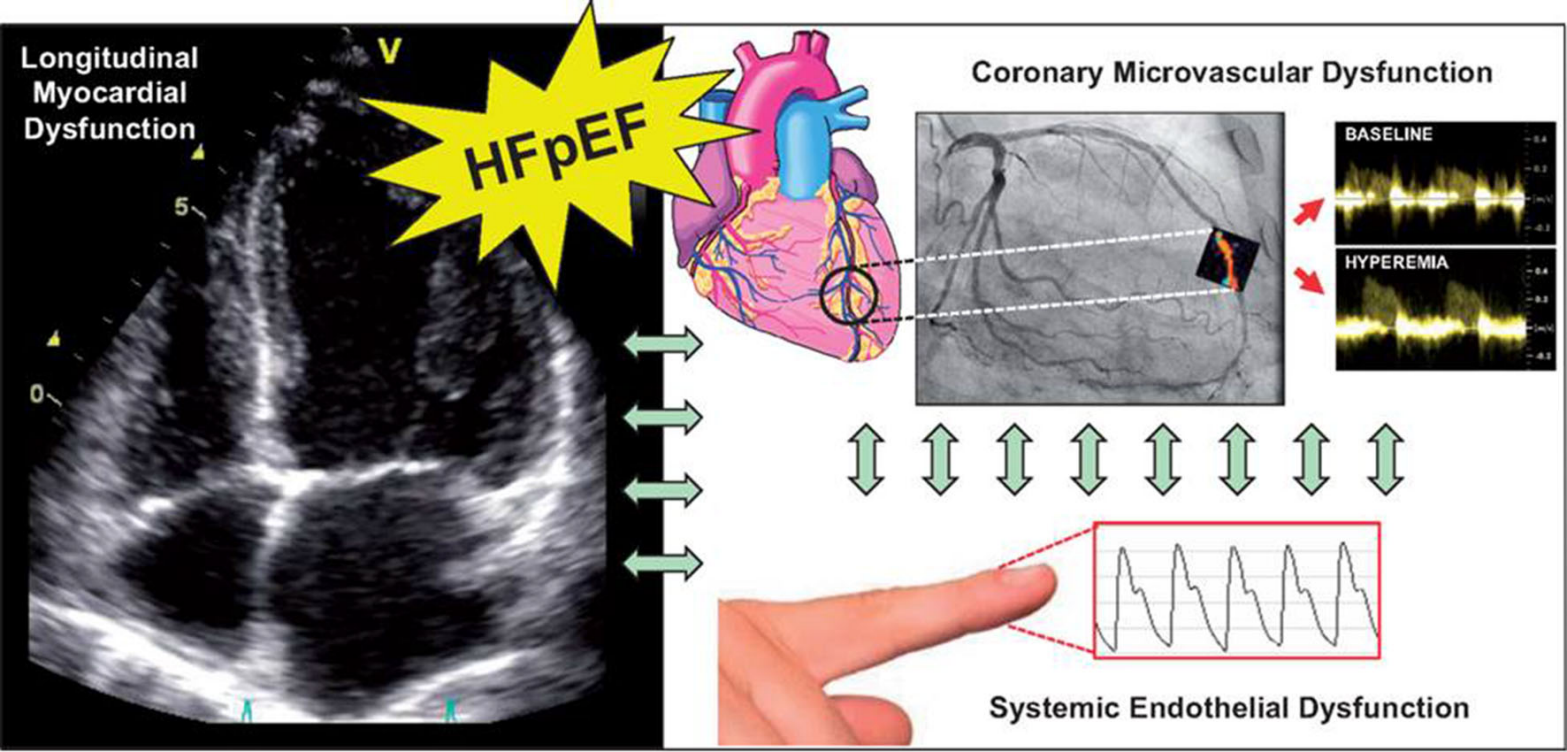
PROMIS-HFpEF study algorithm. In patients diagnosed with heart failure with preserved left ventricular ejection fraction (HFpEF) and nonsignificant coronary artery disease, coronary microvascular dysfunction was assessed based on quantification of coronary flow reserve with adenosine stress transthoracic Doppler echocardiography. In addition, conventional and speckle-tracking echocardiography was used to evaluate left and right ventricular systolic function. Peripheral arterial tonometry was used to measure systemic endothelial dysfunction. Reproduced with permission from Shah et al^1^

Coronary microvascular dysfunction as mechanism underlying the pathophysiology of heart failure with preserved left ventricular ejection fraction (LVEF) was evaluated in the prospective, multicenter PROMIS-HFpEF (PRevalence Of Microvascular dySfunction in Heart Failure with Preserved Ejection Fraction) trial.^1^ Coronary flow reserve (CFR) was measured with adenosine stress transthoracic echocardiography in 202 patients with heart failure and preserved LVEF. In addition, left and right ventricular systolic functions were assessed with conventional and speckle tracking echocardiography and systemic endothelial function was assessed by peripheral arterial tonometry (Figure 1). Three quarters of the patients had coronary microvascular dysfunction (defined by a CFR < 2.5). Patients with coronary microvascular dysfunction were more frequently active smokers (70% vs 43%), had more frequently atrial fibrillation (58% vs 35%), and presented significantly higher levels of NT-proBNP (1050 [396-1930] vs 597 [190-1410] pg/mL; *P* = 0.004) and troponin T (14.0 [10.0-25.6] vs 10.0 [10.0-16.4] ng/mL; *P* = 0.002) compared to patients with preserved microvascular coronary function. Despite similar LVEF, patients with coronary microvascular dysfunction showed more impaired left ventricular (LV) systolic function as assessed with tissue Doppler velocity (s′) (6.3 ± 1.6 vs 7.3 ± 2.1 cm/s; *P* < 0.001) and speckle-tracking global longitudinal strain (GLS) (15.7 ± 3.5 vs 17.0 ± 3.5%; *P* = 0.023) compared with their counterparts. In addition, patients with coronary microvascular dysfunction showed more impaired right ventricular longitudinal strain (21.6 ± 5.2 vs 23.3 ± 5.1%; *P* = 0.005) and lower reactive hyperemia index as a measure of systemic endothelial function. These results suggest that coronary microvascular dysfunction may be an important pathophysiological mechanism of heart failure with preserved LVEF and may set the basis to develop effective therapies that target this mechanism.

Cardiac amyloidosis is characterized by diffuse LV hypertrophy and on speckle tracking analysis, the LV GLS is characterized by a typical apical sparing (with more preserved values of longitudinal strain in the apex, while mid and basal segments show severe impaired strain values). This LV GLS pattern is associated with poor outcomes.^2^ Diffuse LV hypertrophy can be observed in other diseases (hypertension, sarcomeric mutations…) and the prevalence and prognostic implications of relative apical sparing LV longitudinal strain in those other etiologies of LV hypertrophy are unknown. In 399 patients with diffuse LV hypertrophy and no significant coronary artery disease (CAD), Saito et al^3^ showed that relative apical sparing of LV longitudinal strain was present in 10% of patients. During a median follow-up of 4.8 years, 50 patients presented with major adverse cardiovascular events (MACE) (cardiac death, heart failure or acute myocardial infarction). The pattern of relative apical sparing of LV longitudinal strain was more frequently observed among patients presenting with an event. A relative apical sparing LV longitudinal strain index (ratio between the value of apical longitudinal strain and the average longitudinal strain of basal and mid ventricular segments) > 0.6 had incremental prognostic value over clinical and conventional echocardiographic parameters to predict the outcome. These results extend the evidence on the prognostic relevance of relative apical sparing LV longitudinal strain to other causes of LV hypertrophy.

A novel method that integrates blood pressure measurements and LV GLS provides noninvasive LV pressure-strain loops and permits calculation of global and segmental myocardial work in various cardiac diseases. Edwards et al^4^ estimated global and regional myocardial work and wasted work in 10 controls, ten patients with nonischemic cardiomyopathy and 14 patients with ischemic cardiomyopathy. Patients with ischemic and nonischemic cardiomyopathies had significantly lower values of myocardial work compared with controls. In addition, patients with nonischemic cardiomyopathy showed significantly wasted work in the septal wall compared with the lateral wall, suggesting enhanced systolic LV lengthening with segmental shortening during isovolumic relaxation, which can be a target for cardiac resynchronization therapy. In contrast, ischemic patients did not show regional differences in wasted work. This technology was also applied to differentiate physiological from pathological causes of LV hypertrophy in 20 controls, 24 professional athletes and ten patients with nonischemic cardiomyopathy.^5^ Patients with nonischemic cardiomyopathy showed the largest LV mass (317 ± 89 g) followed by the professional athletes (203 ± 57 g) and controls. However, in terms of global myocardial work, nonischemic cardiomyopathy patients showed the most impaired values (723 ± 431 mmHg%) whereas the professional athletes showed the highest values (1931 ± 485 mmHg%). Therefore, the etiology of LV hypertrophy is an important determinant of the global LV myocardial work.

Three-dimensional (3D) transesophageal echocardiography (TEE) is key in the evaluation of patients referred for transcatheter aortic valve replacement, particularly when the anatomy of the aortic valve is bicuspid. In 104 patients with bicuspid aortic valve stenosis, who were evaluated for transcatheter aortic valve replacement but who were eventually treated with surgical aortic valve replacement, Dr Wang^6^ evaluated the agreement between 2-dimensional (2D) transthoracic echocardiography, 3D TEE, and computed tomography (CT) to size the aortic annulus. The reference standard was intraoperative sizing of the aortic annulus. Measurements based on CT and 3D TEE had the best agreement with intraoperative sizing of the aortic annulus. However, the agreement between 3D TEE and intraoperative sizing was negatively influenced by the presence of heavy calcifications of the aortic valve.

Finally, the use of machine learning in echocardiography provides important insights. From the national echocardiographic database of Australia (NEDA), a vendor-agnostic cloud-based database containing echocardiographic data from ten Australian laboratories, Murphy et al^7^ evaluated the prognostic value of left atrial (LA) volume index and LA anteroposterior diameter. Data from 352,844 individuals with a mean follow-up of 5.4 years/person were analyzed. A total of 63,142 deaths were recorded. Left atrial volume index (LAVI) showed the strongest association with all-cause mortality. Individuals with a LAVI > 42 mL/m^2^ (upper quintile) had the highest age- and sex-adjusted risk profile (hazard ratio [HR] 1.9 compared with patients within the lowest quintile [LA volume index < 22 mL/m^2^]). The analysis based on deciles of LA volume index showed that the increase in mortality risk began with the LA volume index decile of 29-31 mL/m^2^ (which is close to the cut-off value proposed by current recommendations to define pathologic dilation of the LA).^8^

## Nuclear Imaging

**Figure 2:**
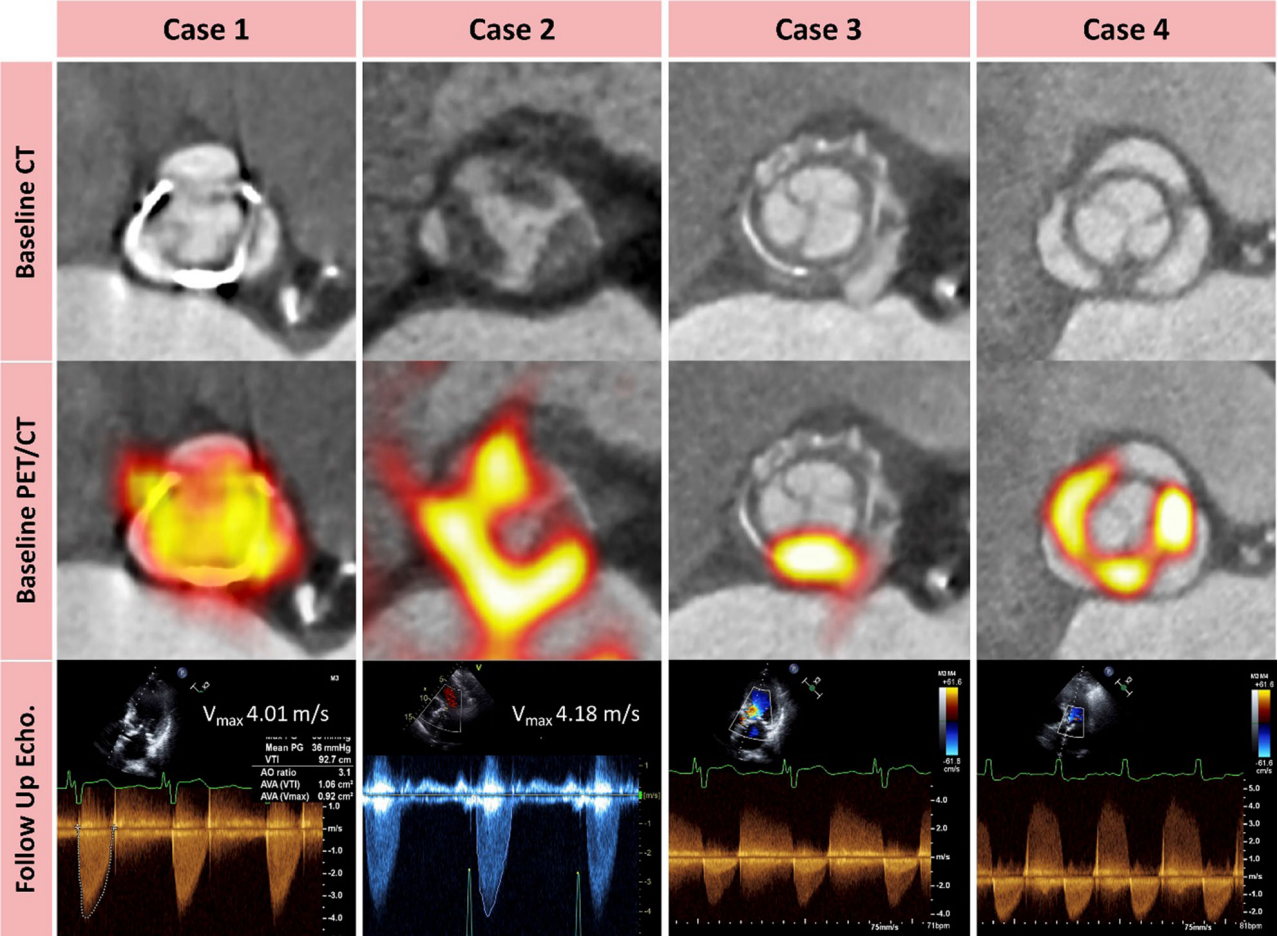
^18^F-fluoride positron emission tomography and computed tomography predicting deterioration in bioprosthetic valve function. Cases 1-4 depict the role of ^18^F-fluoride positron emission tomography (PET) and computed tomography (CT) in predicting new bioprosthetic valve dysfunction. None of the patients had known bioprosthetic degeneration at enrolment. Contrast-enhanced CT images in the short axis of the valve (*top row*) show no clear structural CT changes in case 1, non-calcific leaflet thickening in case 2 and circumferential pannus in cases 3 and 4. Hybrid ^18^F-fluoride PET-CT images (*middle row*) show high intensity 18F-fluoride uptake in each valve. Doppler echocardiography (*bottom row*) shows the development of new valve dysfunction during follow-up with progression to hemodynamically severe stenosis in cases 1 and 2, and new prosthetic regurgitation in cases 3 and 4. Image courtesy of T. Cartridge

Cartlidge et al^9^ used 18F-fluoride positron emission tomography (PET) to predict valvular degeneration of bioprosthetic aortic valves. The hypothesis was based on ex vivo experiments in 15 explanted degenerated bioprosthetic valves, which all showed intense 18F-fluoride uptake coregistered to areas of calcification, fibrosis, thrombosis, and disruption of the collagen structure on micro-CT and on histopathology. In the in vivo study, the investigators performed 18F-fluoride PET in 6 patients with aortic bioprosthetic valve dysfunction (cohort 1) and in 71 patients without any known prosthesis dysfunction (cohort 2). Patients were followed up 2 years after PET/CT with echocardiography and clinical assessment. In cohort 1, all patients exhibited increased 18F-fluoride uptake on PET and leaflet abnormalities on CT. Interestingly, in cohort 2, 27 (38%) patients had increased 18F-fluoride uptake with a mean target-to-background (TBR) ratio of 1.55. Patients with increased 18F-fluoride uptake demonstrated a > 10-fold increase in mean gradient over 12 months compared to those without increased uptake (Figure 2). On multivariable analysis, baseline 18F-fluoride uptake was the only independent predictor of deterioration in bioprosthetic function, and all patients with TBR values ≥ 2.5 had evidence of overt bioprosthetic failure within 1 year of imaging. The findings of this study suggest that 18F-fluoride PET may be a powerful tool to detect early bioprosthetic valve degeneration and guide subsequent patient management.

Machine learning principles (e.g. deep artificial neural networks = deep learning) are raising increasing attention as innovative approaches to explore and learn complex patterns within imaging data and improve human interpretation of large data clusters. Juarez-Orozco et al^10^ used a pretrained very deep convolutional neural network modified through transfer learning for the identification of patients who experience MACE based on direct image processing of PET myocardial perfusion polar maps. For this purpose, polar maps of myocardial perfusion reserve from 1199 patients (625 female, mean age 68 years) undergoing 13N-ammonia PET were analyzed. Deep learning was built through transfer learning by obtaining the architecture of the open source pretrained ResNet50 convolutional neural network and replacing the last layer and associated weights with a new layer specialized for the classification of patients who experienced or not MACE using a 9:1 training to testing ratio and 5-fold cross validation. Deep learning demonstrated an overall cross-validated validation accuracy of 77% with a corresponding precision and recall of 90% and 72%, respectively, and a specificity of 87% for identifying patients who experienced a MACE on follow-up. The positive likelihood ratio was 5.51, while the negative likelihood ratio was 0.32. The authors conclude that deep learning may offer additional clinical value in the identification of patients that will present MACE on follow-up.

Hybrid 18F-fluorodeoxyglucose (FDG) PET and magnetic resonance imaging (MRI) permit differentiation between myocardial scar from fibrosis associated with inflammation. Spinelli et al^11^ explored the value of FDG PET/MRI to detect early signs of myocardial inflammation in patients with Anderson-Fabry disease. Cardiac FDG PET/MRI and transthoracic strain echocardiography were performed in 24 heterozygous females carrying α-galactosidase A mutation and without LV hypertrophy. Thirteen patients showed focal FDG uptake (defined by a coefficient of variation in segmental FDG uptake > 0.17), including 2 patients with late gadolinium enhancement on MRI. Patients with a coefficient of variation > 0.17 had worse LV GLS compared to those with coefficient of variation ≤ 0.17 (− 18.5 ± 2.7% vs − 22.2 ± 1.8%, *P* = 0.024). The authors conclude that focal FDG uptake is an early sign of disease-related myocardial damage in patients with Anderson-Fabry disease and is associated with impaired LV longitudinal function. Moreover, their findings support the notion that inflammation plays an important role in glycosphingolipids storage disorders.

Keller et al^12^ report feasibility and safety profiles of pharmacological stress testing with the selective A2A receptor agonist regadenoson in 5151 consecutive patients undergoing 99m-TcTetrofosmin myocardial perfusion scintigraphy. The most frequent side effects were shortness of breath (66.2%), headache (21.3%), feeling of warmth (20.7%), feeling of pressure in the chest (18.1%) and in the “stomach” (17.4%). A new first degree atrioventricular block was observed in 0.1%. In four patients (0.08%), a severe symptomatic bradycardia or even life-threatening asystole occurred which could be immediately interrupted by administration of aminophyllin and atropine. These side effects were more often observed in patients with preexistent first degree atrioventricular block. This prospective registry demonstrates that regadenoson for pharmacological stress tests is generally well tolerated with frequent but harmless and transient side effects. Although very rare, life-threatening asystole or severe bradycardia (0.08%) may occur but can be rapidly treated with aminophyllin and atropine.

## Cardiovascular Magnetic Resonance Imaging

**Figure 3:**
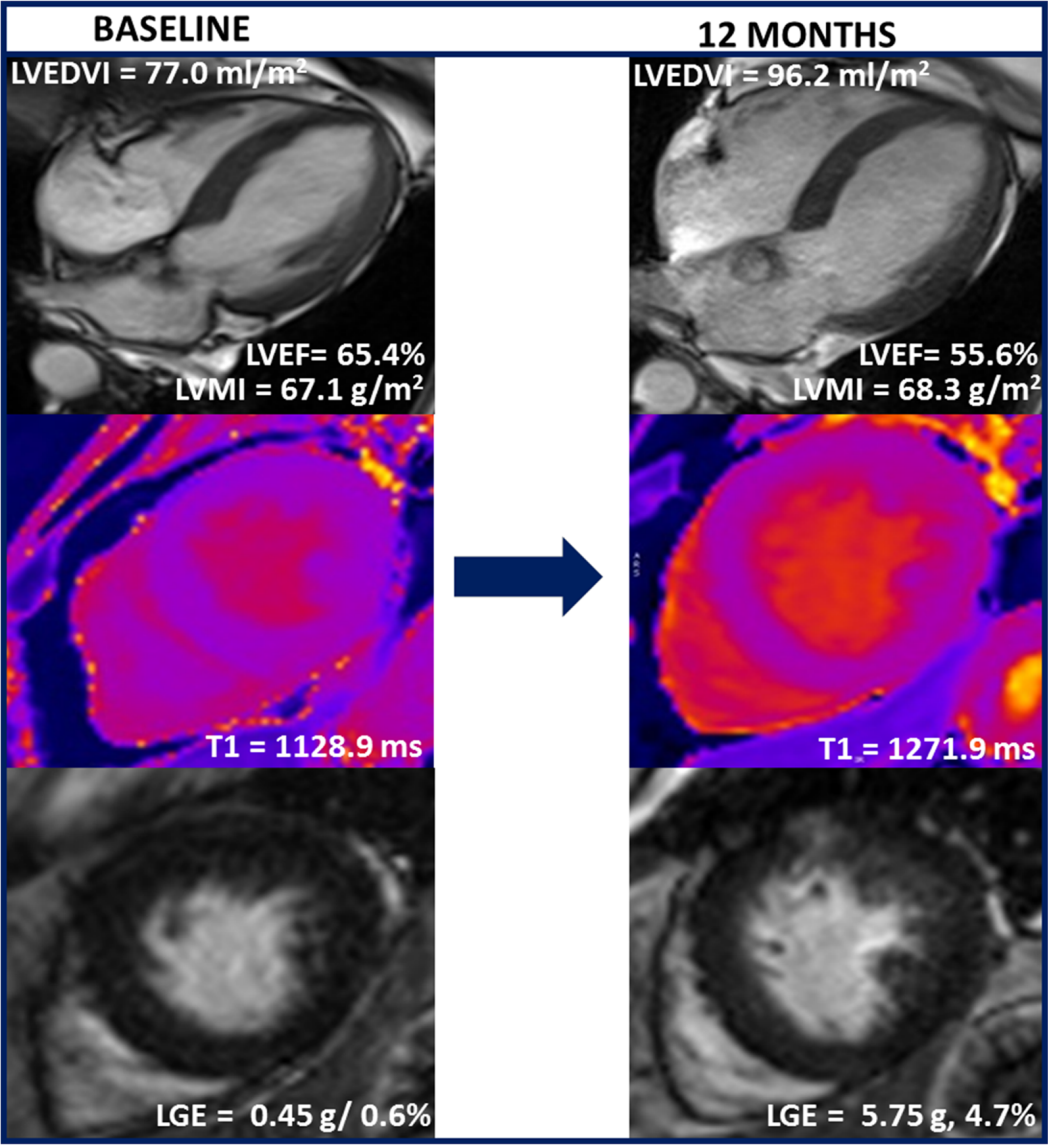
Short-term adverse cardiac remodeling in asymptomatic moderate and severe aortic stenosis by cardiovascular magnetic resonance. A representative patient undergoing cardiovascular magnetic resonance at baseline at 12-month follow-up. There is a progressive increase in left ventricular end-diastolic volume (LVEDV) and left ventricular mass index (LVMI), reduction in left ventricular ejection fraction (LVEF) and increases in myocardial fibrosis by both native T1 mapping and late gadolinium enhancement (LGE) in the interval of 12 months, whereas the indexed aortic valve area remained unchanged. These changes suggest adverse cardiac remodeling despite no further reduction in indexed aortic valve area. Reproduced with permission from Singh et al^13^

Watchful waiting in asymptomatic patients with severe aortic stenosis and preserved LVEF is currently recommended. However, the pressure overload imposed to the LV by the stenotic valve can lead to irreversible myocardial fibrosis which has shown to portend poor prognosis even after aortic valve intervention. Singh et al^13^ investigated whether adverse LV remodeling progresses in the short term in 44 asymptomatic patients with moderate-to-severe aortic stenosis (83.4% male, indexed aortic valve area 0.54 ± 0.15 cm^2^/m^2^) included in the PRIMID-AS (PRognostic Importance of MIcrovascular Dysfunction in Aortic Stenosis) study. Cardiovascular magnetic resonance (CMR) imaging was performed at baseline and 12-month follow-up. The authors observed a significant increase in indexed LV end-diastolic volume (from 90.7 ± 22.0 to 94.5 ± 23.1, *P* = 0.007) and indexed LA volume (from 52.9 ± 11.3 58.6 ± 13.6, *P* < 0.001), reduction in LVEF (from 57.9 ± 4.6% to 55.6 ± 4.1%, *P* = 0.001) and impairment in LV diastolic function (longitudinal diastolic strain rate from 1.06 ± 0.24 s^−1^ 0.99±0.24 s^−1^, *P* = 0.026) despite no change in the indexed aortic valve area. In addition, there was a significant reduction in rest and stress myocardial blood flows and increases in myocardial fibrosis both on native T1 mapping and late gadolinium enhancement (LGE) imaging (Figure 3). These findings demonstrate unequivocal progression of adverse cardiac remodeling in asymptomatic moderate-severe aortic stenosis in 12 months, emphasizing the need of improved patient risk-stratification tools and potential need for earlier intervention.

Boldrini et al^14^ evaluated the accuracy of noncontrast CMR for clinical diagnosis of cardiac amyloidosis in a large multicenter prospective study of 868 patients with suspected systemic amyloidosis. In addition to comprehensive clinical investigations, the imaging protocol included native (noncontrast) T1 mapping and LGE imaging. The final diagnosis was light-chain cardiac amyloidosis in 222, transthyretin cardiac amyloidosis in 214, and no cardiac amyloidosis in 427 patients. Native T1 was higher in patients with light-chain or transthyretin cardiac amyloidosis compared with patients without cardiac amyloidosis. The diagnostic accuracy of native T1 to identify patients with cardiac amyloidosis was excellent (area under the curve 0.93). A native T1 < 1036 ms was the most sensitive value (99% sensitivity) to exclude cardiac amyloidosis while a native T1 > 1164ms was the most specific (99% specificity) to diagnose cardiac amyloidosis. Based on these results, the authors proposed a diagnostic algorithm with native T1 mapping to all patients with suspected cardiac amyloidosis, whereas contrast administration and LGE imaging would be reserved for patients with native T1 mapping between 1036 and 1164ms.

Feature-tracking CMR is a novel technique that permits assessment of myocardial deformation on cine CMR images. Using this methodology, Podlesnikar et al^15^ evaluated the effects of early metoprolol on the LV systolic function in 214 patients with anterior ST-segment myocardial infarction treated with primary percutaneous coronary intervention. The patients were included in the METOCARD-CNIC (Effect of Metoprolol in Cardioprotection During an Acute Myocardial Infarction) trial and randomized to early intravenous metoprolol vs standard care. CMR was performed 5-7 days after reperfusion, and LV global circumferential strain (GCS) and GLS were measured using feature tracking. During a median follow-up of 2 years, 22 MACE (composite of death, heart failure readmissions, reinfarction, and malignant ventricular arrhythmias) occurred. Patients with LV GCS *>* − 13.2% or with LV GLS *>* − 11.5% (more impaired LV systolic function) who did not receive early intravenous metoprolol showed the highest cumulative event rates compared with the patients who received metoprolol or had more preserved LV systolic function. These results underscore the role of feature-tracking CMR for detailed risk stratification of patients with ST-segment elevation myocardial infarction.

Phase-contrast cine MRI of the coronary sinus is a noninvasive method to quantify CFR which has been associated with prognosis in patients with suspected CAD. In 163 patients with diabetes mellitus, Kato et al^16^ assessed CFR during adenosine triphosphate infusion and at rest using phase-contrast cine MRI. CFR was calculated from myocardial blood flows at stress and rest. During a mean follow-up period of 3.7 years, 20 MACEs were identified, and patients with CFR < 2.0 had worse prognosis than those with preserved CRF > 2.0. On multivariable Cox regression analysis, CFR < 2.0 was independently associated with MACE (HR 11.29, *P* < 0.001). The study demonstrated that a noninvasive CFR assessment by phase-contrast cine MRI could represent a novel risk stratification tool in patients with diabetes mellitus.

## Cardiac Computed Tomography

The 5-year follow-up results of the Scottish Computed Tomography of the Heart (SCOT-HEART) trial were presented at this ESC congress in Munich.^17^ Patients with stable chest pain who were referred to a cardiology clinic were randomized to coronary computed tomography angiography (CTA) in addition to standard care (n = 2073) versus standard care alone (n = 2073). The initial results of this trial showed that coronary CTA clarified the diagnosis and altered the subsequent management of the patients at short-term follow-up.^18^ Specifically, coronary CTA resulted in cancelations of a significant number of functional tests, increase the number of invasive coronary angiography and led to an increase in the prescription of preventive and antianginal medications. At 5-year follow-up, coronary CTA and the associated changes in treatment resulted in significantly lower rates of death from coronary heart disease and nonfatal myocardial infarction compared with standard care (2.3% vs 3.9%; HR 0.59; *P* = 0.004). Importantly, the rates of invasive coronary angiography and coronary revascularization were similar at 5 years in both arms.

In the EPICardial Adipose Tissue in HEART Diseases (EPICHEART) study,^19^ 574 patients with severe aortic stenosis referred to surgical aortic valve replacement were included and the influence of epicardial adipose tissue (EAT) volume on coronary artery calcification was evaluated. Using CT, the EAT volume was measured. In addition, quantitative proteomics of the thoracic fat (including EAT, mediastinal, and subcutaneous adipose tissue) were performed. EAT volume was not associated with the extent of coronary artery calcification on CT. However, quantitative proteomics of the EAT showed that patients with coronary artery calcification had upregulation of pro-calcifying annexins, fatty acid-binding transporters, and inflammatory signaling proteins whereas fetuin-A and antioxidant enzymes were downregulated. In EAT, pro-calcifying annexins regulation was positively correlated with coronary artery calcium. This imbalance of pro-calcifying, pro-inflammatory and lipid transporter mediators was not observed in patients without coronary artery calcification. These results indicate that the biological characteristics of the EAT rather than the volume of EAT exerts an influence on the coronary atherosclerotic process. The Cardiovascular Risk Prediction using Computed Tomography (CRISP-CT) study provided further insight into the prognostic value of perivascular fat characteristics.^20^ The signals released by the inflamed coronary atherosclerotic plaques exert an effect on the perivascular adipose tissue inhibiting local adipogenesis. The changes in the composition of the perivascular fat around the inflamed coronary arteries result in a change in attenuation of coronary CTA (from more negative Hounsfield unit [HU] values to less negative) which can be quantified with the perivascular fat attenuation index. On a derivation cohort of 1872 participants with a median follow-up of 72 months and a validation cohort of 2040 participants with a median follow-up of 54 months, the perivascular fat attenuation index values around the proximal right coronary artery and left anterior descending artery were measured on CTA and correlated with the occurrence of all-cause and cardiac mortality. A value of perivascular fat attenuation index of − 70.1 HU or higher (less negative) was associated with 2.5-fold increase in all-cause mortality and 9-fold increase in cardiac mortality in the derivation cohort. This cutoff value was confirmed in the validation cohort (HR 3.69, *P* < 0.001 for all-cause mortality and HR 5.62, *P* < 0.001 for cardiac mortality). Therefore, perivascular fat attenuation index reflects coronary inflammation and could guide preventive therapies in primary prevention and intensify secondary prevention in patients with CAD.

**Figure 4:**
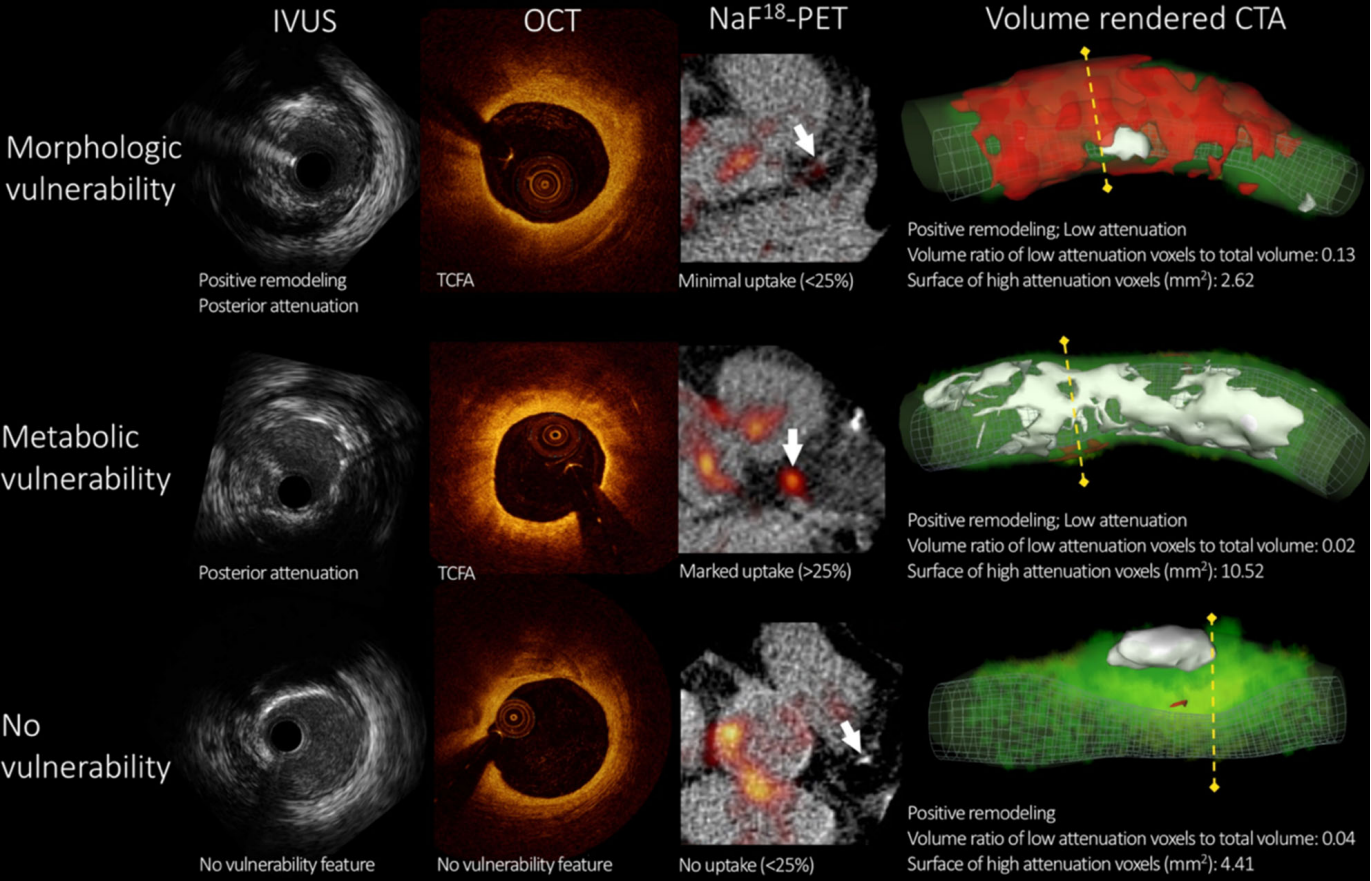
Intravascular ultrasound, optical coherence tomography, 18F-sodium fluoride-PET, and volume-rendered coronary computed tomography angiography images of three representative coronary lesions to assess plaque vulnerability. Morphologic vulnerability was defined as the presence of positive remodeling and posterior attenuation on intravascular ultrasound (IVUS) and the presence of thin-cap fibroatheroma (TCFA) or intraplaque microvessels or macrophage infiltration on optical coherence tomography (OCT). Metabolic vulnerability was defined as any plaque showing > 25% uptake on 18F-sodium fluoride-PET (NaF^18^-PET) images. *Upper row* depicts a plaque showing morphologic characteristics of vulnerability. *Middle row* shows a lesion with metabolic characteristics of vulnerability. *Bottom row* displays a plaque showing neither morphologic nor metabolic characteristics of vulnerability. Image courtesy of M. Kolossvary. CTA, computed tomography angiography

Another pioneering study describing coronary CTA radiomics to identify novel imaging biomarkers of plaque vulnerability was presented by Kolossvary et al^21^ Coronary CTA radiomics provides multiple quantitative metrics that describe the heterogeneity and spatial complexity of coronary lesions, resulting in big-data datasets, where each abnormality is characterized by hundreds of different parameters. The investigators aimed to assess the discriminatory power of coronary CT radiomics to identify morphologic and/or metabolic parameters of plaque vulnerability. A total of 44 plaques were analyzed in 25 patients using intravascular ultrasound (IVUS), optical coherence tomography (OCT), 18F-fluoride-PET, and coronary CTA. Beyond conventional qualitative and quantitative metrics, the investigators calculated 935 radiomic parameters of each plaque. Morphologic vulnerability was defined by IVUS and OCT, whereas metabolic vulnerability was defined by 18F-fluoride-PET (Figure 4). The best conventional CT metric resulted in an area under the curve value close to random (0.52), while the best radiomic feature had a good diagnostic accuracy (area under the curve = 0.74) to identify morphologic plaque vulnerability. Remarkably, radiomic features also outperformed conventional CT metrics to identify metabolic plaque vulnerability (area under the curve: 0.87 vs 0.65, respectively). These results are promising since CT radiomics can capture both morphologic and metabolic high-risk plaque characteristics. Additional prospective studies evaluating how CT radiomics may help to guide therapy are needed.

As coronary CTA is becoming a widely available imaging technique and one of the first-line tests used in patients with suspected CAD, the radiation dose exposure is highly relevant. Hausleiter et al. presented the results of the Prospective Multicenter Registry on RadiaTion Dose Estimates of Cardiac CT AngIOgraphy iN Daily Practice (PROTECTION-VI) study that evaluated the radiation dose and the utilization of dose-saving strategies for cardiac CTA.^22^ The study conducted in 61 hospitals from 32 countries prospectively enrolled 4502 patients undergoing cardiac CTA during one calendar month in 2017. The coronary CTA data were analyzed in a central core-lab and compared with a similar dose survey performed in 2007. The median dose-length product (DLP) of coronary CTA was 195 mGy*cm, 78% lower than the DLP reported in the 2007 survey (*P* < 0.001). This reduction in radiation dose was not associated with the increase in the number of nondiagnostic coronary CTA (1.7% in 2007 vs 1.9% in 2017 surveys, *P* = 0.55). This large international radiation dose survey demonstrates considerable reduction of radiation exposure in coronary CTA during the last decade and underlines the importance of training and adaptation of contemporary cardiac scan protocols.

## Abbreviations

CAD: Coronary artery disease
CMR: Cardiac magnetic resonance
CT: Computed tomography
FDG: 18F-Fluorodeoxyglucose
GLS: Global longitudinal strain
LGE: Late gadolinium enhancement
LV: Left ventricular
LVEF: Left ventricular ejection fraction
PET: Positron emission tomography
TEE: Transesophageal echocardiography
